# Patient Comorbidities Associated With Acute Infection After Open Tibial Fractures

**DOI:** 10.5435/JAAOSGlobal-D-22-00196

**Published:** 2022-09-23

**Authors:** Augustine M. Saiz, Dustin Stwalley, Philip Wolinsky, Anna N. Miller

**Affiliations:** From the Department of Orthopaedic Surgery, UC Davis Health, Sacramento, CA (Dr. Saiz, Jr and Dr. Wolinsky); the Department of Medicine, Washington University in St. Louis, St. Louis, MO (Mr. Stwalley); and the Department of Orthopaedic Surgery, Washington University in St. Louis, St. Louis, MO (Dr. Miller).

## Abstract

Open tibial shaft fractures are high-risk injuries for developing acute infection. Prior research has focused on injury characteristics and treatment options associated with acute inpatient infection in these injuries without primary analysis of host factors. The purpose of this study was to determine the patient comorbidities associated with increased risk of acute infection after open tibial shaft fractures during initial hospitalization.

**Methods:**

A total of 147,535 open tibial shaft fractures in the National Trauma Data Bank from 2007 to 2015 were identified that underwent débridement and stabilization. Infection was defined as a superficial surgical site infection or deep infection that required subsequent treatment. The International Classification of Diseases, ninth revision codes were used to determine patient comorbidities. Comparative statistical analyses including odds ratios (ORs) for patient groups who did develop infection and those who did not were conducted for each comorbidity.

**Results:**

The rate of acute inpatient infection was 0.27% with 396 patients developing infection during hospital management of an open tibial shaft fracture. Alcohol use (OR, 2.26, 95% confidence interval [CI], 1.73-2.96, *P* < 0.0001), bleeding disorders (OR, 4.50, 95% CI, 3.13-6.48, *P* < 0.0001), congestive heart failure (OR, 3.25, 95% CI, 1.97-5.38, *P* < 0.0001), diabetes (OR, 1.73, 95% CI, 1.29-2.32, *P* = 0.0002), psychiatric illness (OR, 2.17, 95% CI, 1.30-3.63, *P* < 0.0001), hypertension (OR, 1.56, 95% CI, 1.23-1.95, *P* < 0.0001), obesity (OR, 3.05, 95% CI, 2.33-3.99, *P* < 0.0001), and chronic obstructive pulmonary disease (OR, 2.09, 95% CI, 1.51-2.91, *P* < 0.0001) were all associated with increased infection rates. Smoking (OR, 0.957, 95% CI, 0.728-1.26, *P* = 0.722) and drug use (OR, 1.11, 95% CI, 0.579-2.11, *P* = 0.7607) were not associated with any difference in infection rates.

**Discussion:**

Patients with open tibial shaft fractures who have congestive heart failure, bleeding disorders, or obesity are three to 4.5 times more likely to develop an acute inpatient infection than patients without those comorbidities during their initial hospitalization. Patients with diabetes, psychiatric illness, hypertension, or chronic obstructive pulmonary disease are 1.5 to 2 times more likely to develop subsequent infection compared with patients without those comorbidities. Patients with these comorbidities should be counseled about the increased risks. Furthermore, risk models for the infectious complications after open tibial shaft fractures can be developed to account for this more at-risk patient population to serve as modifiers when evaluating surgeon/hospital performance.

**Conclusion:**

Patient comorbidities are associated with increased risk of acute inpatient infection of open tibial shaft fractures during hospitalization.

Open tibial shaft fractures are relatively common long bone injuries.^1,2^ These fractures are at high risk of acute infection even with proper antibiotic and surgical treatment.^3^ The development of acute infection in open tibial shaft fractures has substantial negative effects on patients' outcomes and economic healthcare costs.^4-6^ As such, the ability to predict which open tibial shaft fracture patients are at increased risk of infection and could benefit from treatment strategies to decrease that risk is an important area of research in orthopaedic trauma.

Previous studies investigating acute infection in open tibial shaft fractures have mostly focused on intrinsic injury characteristics and extrinsic variables such as antibiotic prophylaxis, time to débridement, soft-tissue management, and surgical management.^3,7-22^ These factors (fracture type, severity of injury, soft-tissue compromise, and antibiotic and surgical timing) all play an important role in the development of acute infection in open tibial shaft fractures. However, the effect of intrinsic patient characteristics on the development of acute infection has not been studied as extensively. These nonmodifiable host factors may affect clinical outcomes beyond the control of the surgeon, even when using the appropriate treatment algorithms. The few studies to have included these analyses are primarily based on single-institution data, are often underpowered or not generalizable, and also include closed fractures.^13,23^

The goal of this study was to determine the patient comorbidities associated with increased risk of acute infection in open tibial shaft fractures. Our investigation aims to provide surgeons with awareness of which patients are at higher risk and to define a patient population to serve as modifiers when evaluating surgeon/hospital performance.

## Methods

Data from the National Trauma Data Bank (NTDB) from 2007 to 2015 were examined. The NTDB was established by the American College of Surgeons in 1997 and represents the largest collection of trauma data in the United States.^24^ The data are obtained from level I, II, III, and IV trauma centers in the United States representing a national probability sample. Seven hundred forty-seven trauma centers have contributed more than seven million cases. The NTDB contains specific variables of interest to trauma for an immense number of patients from all payors. Trained analysts abstract data from the medical record, using explicit algorithms. The NTDB is a deidentified database and undergoes quality screening. As such, our study received an expediated review and approval from our institutional review board.

Study inclusion criteria were patients older than 18 years with a diagnosis code for an open fracture of the tibia with or without a fibular fracture. Exclusion criteria were patients with multiple fractures to ipsilateral extremity (eg, ankle fracture, plateau) or patients with incomplete data. The International Classification of Diseases, ninth revision (ICD-9) codes for the tibial shaft fractures included 823, followed by 30 (open fracture of tibia shaft alone) and 32 (open fracture of tibia shaft with fibula). ICD-9 codes for infection included skin and soft-tissue infections (020.1, 021.0, 022.0, 032.85, 035, 039.0, 039.3-040.3, 040.42, 040.81, 078.3, 082.0-088.9, 098.50, 567.31, 675.00-675.14, 675.80-675.94, 680.0-685.0, 686.00-686.9, 705.83, 727.89, 728.0, 728.86), surgical site infections (996.60-996.69, 998.51, 998.59), and bone infections (003.23, 003.24, 026.1, 036.82, 098.50-098.59, 376.03, 513.1, 519.2, 711.00-711.09, 711.90-711.99, 730.00-730.39, 730.80-730.99).

The data set for patients with open fractures of the tibia with and without acute infection during initial hospitalization was analyzed. Two groups of patients were created: group 1—those with open tibial shaft fractures and acute infection and group 2—those with an open tibial shaft fracture that did not have an acute infection. External cause of injury codes (E code), Injury Severity Scores, and comorbidities were recorded for both groups. Treatments, including fracture fixation, skin grafting, and surgical flap coverage, were also recorded. The primary outcome was comorbidities associated with the presence of an acute infection. Infection was defined as either a surgical site infection or a deep infection that required subsequent treatment. Patient comorbidities were determined from ICD-9 codes. Secondary outcomes included patient demographics, fracture characteristics, and treatment variables.

Percentages and relative risks with 95% confidence interval (CI) were the outcome data. The open tibial shaft fractures with infection and without infection groups were compared regarding clinical characteristics using the Student *t*-test for continuous symmetrically distributed variables, the Mann-Whitney test for continuous asymmetrically distributed variables, and the chi square test. Statistical significance was set at a two-tail *P* value of 0.05. All analyses were conducted using SAS. Logistic regression was not used to adjust the association for specific demographics, injury/fracture characteristics, or comorbidities owing to a very few outcomes for our planned model.

## Results

We identified 147,535 patients with an open tibial shaft fracture who underwent débridement and stabilization. Of those, 396 developed an infection during acute hospital management for an infection rate of 0.27%.

### Demographics/Mechanisms of Injury/Fracture Characteristics

Patients who developed an acute inpatient infection tended to be older (40 years or older) (odds ratio [OR], 1.36, 95% CI, 1.11-1.67, *P* < 0.003), to be non-White (OR, 1.33, 95% CI, 1.09-1.64, *P* < 0.008), and to have a higher Injury Severity Score (greater than/equal to 18) (OR, 2.09, 95% CI, 1.68-2.60, *P* < 0.001) than those without an infection (Table 1). A variety of mechanisms of injury (MOIs) were associated with an increased risk of developing an infection (Table 2). Most had a higher energy MOI consisting of motor vehicle collisions (OR, 1.66, 95% CI, 1.34-2.05, *P* < 0.0001).

**Table 1:** Demographics

Factor	Infection, n (%)	No Infection, n (%)	*P* Value	Odds Ratio	95% Confidence Interval
Sex, female	102 (26.3)	43,872 (29.9)	0.12	—	—
Age			0.04	—	—
18-39	148 (37.3)	66,009 (44.9)			
40-54	135 (34.1)	42,715 (29.0)			
55-64	69 (17.4)	21,505 (14.6)			
65-74	28 (7.1)	10,026 (6.8)			
75-84	14 (3.5)	5,298 (3.6)			
85+	2 (0.5)	1,586 (1.1)			
Age, 18-39 vs 40+	148 (37.3)	66,009 (44.9)	**0.003**	**1.36**	**1.11-1.67**
Race			0.02		
White	246 (64.7)	98,380 (70.9)			
African American	86 (22.6)	24,349 (17.6)			
Other	48 (12.6)	16,010 (11.5)			
Race, White vs. non-White	246 (64.7)	98,380 (70.9)	**0.008**	**1.33**	**1.08-1.64**
ISS			**<0.0001**		
0-9	102 (30.2)	65,497 (45.9)			
10-14	82 (24.3)	37,949 (26.6)			
15-19	36 (10.7)	11,430 (8.0)			
20-24	33 (9.8)	7,902 (5.5)			
25-29	43 (12.7)	8,319 (5.8)			
30-34	17 (5.0)	3,961 (2.8)			
35+	25 (7.4)	7,541 (5.3)			
ISS, 18+ vs 0-17	133 (39.4)	33,823 (23.7)	**<0.0001**	**2.09**	**1.68-2.60**

Statistical significance was set of *P* < 0.01. Bold items met that level. ISS = Injury Severity Score

**Table 2:** Mechanism of Injury

Factor	Infection, n (%)	No Infection, n (%)	*P* Value	Odds Ratio	95% Confidence Interval
MVC	263 (66.8)	79,849 (54.8)	**<0.0001**	**1.66**	**1.34-2.05**
Pedestrian	4 (1.0)	1,266 (0.9)	0.75	1.17	0.44-3.14
Gunshot	16 (4.1)	9,524 (6.5)	0.05	0.61	0.37-0.99
Machinery	5 (1.3)	2,085 (1.4)	0.79	0.89	0.37-2.14

Statistical significance was set of *P* < 0.01. Bold items met that level. MVC = motor vehicle collision

### Comorbidities

Alcohol use (OR, 2.26, 95% CI, 1.73-2.96, *P* < 0.0001), bleeding disorders (OR, 4.50, 95% CI, 3.13-6.48, *P* < 0.0001), congestive heart failure (OR, 3.25, 95% CI, 1.97-5.38, *P* < 0.0001), diabetes (OR, 1.73, 95% CI, 1.29-2.32, *P* = 0.0002), psychiatric illness (OR, 2.17, 95% CI, 1.30-3.63, *P* < 0.0001), hypertension (OR, 1.56, 95% CI, 1.23-1.95, *P* < 0.0001), obesity (OR, 3.05, 95% CI, 2.33-3.99, *P* < 0.0001), and chronic obstructive pulmonary disease (COPD) (OR, 2.09, 95% CI, 1.51-2.91, *P* < 0.0001) were all associated with increased infection rates (Table 3). Smoking (OR, 0.957, 95% CI, 0.728-1.26, *P* = 0.722) and drug use (OR, 1.11, 95% CI, 0.579-2.11, *P* = 0.7607) were not associated with any difference in infection rates (Table 3).

**Table 3:** Patient Comorbidities

Factor	Infection, n (%)	No Infection, n (%)	*P* Value	Odds Ratio	95% Confidence Interval
Alcohol use	332 (0.23)	135,587 (91.9)	**<0.0001**	**2.26**	**1.73-2.96**
Bleeding disorders	364 (0.25)	144,322 (97.8)	**<0.001**	**4.50**	**3.13-6.48**
CHF	380 (0.26)	145,259 (98.5)	**<0.0001**	**3.25**	**1.97-5.38**
Diabetes	345 (0.23)	135,565 (91.9)	**0.0002**	**1.73**	**1.29-2.32**
Psychiatric illness	105 (0.15)	66,665 (92.9)	**<0.0001**	**2.17**	**1.30-3.63**
Hypertension	292 (0.20)	119,746 (81.2)	**<0.0001**	**1.56**	**1.23-1.95**
Obesity	315 (0.25)	119,640 (93.4)	**<0.0001**	**3.05**	**2.33-3.99**
COPD	356 (0.24)	139,643 (94.7)	**<0.0001**	**2.09**	**1.51-2.91**
Smoking	335 (0.23)	123,617 (83.8)	0.722	0.957	0.728-1.26
Drug use	112 (0.16)	66,283 (92.4)	0.761	1.11	0.579-2.11

Statistical significance was set of *P* < 0.01. Bold items met that level. CHF = congestive heart failure, COPD = chronic obstructive pulmonary disease

### In-Hospital Treatment Interventions

Patients with open tibial shaft fractures that had an acute inpatient infection were more likely to have external fixation (OR, 2.97, 95% CI, 2.44-3.62, *P* < 0.0001), fasciotomy (OR, 2.45, 95% CI, 1.81-3.33, *P* < 0.0001), skin grafting (OR, 5.98, 95% CI, 4.85-7.38, *P* < 0.0001), and/or surgical flap coverage (OR, 6.73, 95% CI, 5.10-8.88, *P* < 0.0001) done than those with fractures that did not develop an infection (Table 4).

**Table 4:** Hospital Treatments

Factor	Infection, n (%)	No Infection, n (%)	*P* Value	Odds Ratio	95% Confidence Interval
External fixation	188 (47.4)	34,357 (23.4)	**<0.0001**	**2.97**	**2.44-3.62**
Fasciotomy	47 (11.9)	7.665 (5.2)	**<0.0001**	**2.45**	**1.81-3.33**
Skin graft	132 (33.3)	11,354 (7.7)	**<0.0001**	**5.98**	**4.85-7.38**
Surgical flap	60 (15.2)	3,801 (2.6)	**<0.0001**	**6.73**	**5.10-8.88**

Statistical significance was set of *P* < 0.01. Bold items met that level.

## Discussion

Open tibial shaft fractures remain a common, challenging injury for orthopaedic surgeons because of the risk of complications. There has been immense research investigating treatment protocols to decrease the risk of infection, a dire complication due to the open nature of the injury. Through this research, modifiable risk factors have been identified with resulting adaptations in treatment algorithms to improve outcomes. Our study investigated nonmodifiable patient characteristics associated with increased risk of acute infection after treatment of open tibial shaft fractures during initial hospitalization. We determined that patients with congestive heart failure, bleeding disorders, or obesity are three to 4.5 times more likely to develop an infection than patients without those comorbidities. Similarly, patients with diabetes, psychiatric illness, hypertension, or COPD are 1.5 to 2 times more likely to develop subsequent infection compared with patients without those comorbidities.

To the best of our knowledge, this is the largest data set analyzed to determine the nonmodifiable patient risk factors for developing an acute infection after treatment of open tibial shaft fractures. The incidence of acute infection after the treatment of open tibial shaft fractures was 0.27%. An acute infection incidence of 4.3% was reported in 646 open tibia fractures and used the Charlson Comorbidity Index (CCI) without breakdown of specific comorbidities without any difference in infection between those with higher CCI scores and those with lower CCI scores.^3^ Another study of 4,963 tibial shaft fractures showed an overall infection rate of 0.02% within 30 days of ORIF/IMN but different rates of infection between open and closed fractures were not reported.^19^ In that same study though, a higher American Society of Anesthesiologists score, congestive heart failure, and hypertension were associated with acute infection.^19^ In 480 patients with tibial shaft fractures, another study did not identify any patient comorbidities as risk factors for acute infection.^13^ By contrast, our study specifically investigated open tibial shaft fractures and accounted for patient comorbidities granularly as compared with amalgamated scores such as CCI or American Society of Anesthesiologists.

As with most large database studies, the inability for granular analysis and possible coding error are limitations. The surprisingly low infection rate and sample size may be because of missed instances of infection reported. In addition, compared with other studies, this study using the NTDB only captures inpatient complications and infections that often do not present until later in the clinical course. As such, acute versus subacute versus chronic infection cannot be determined using the NTDB. Furthermore, patients with increased comorbidities may be in the hospital longer, hence more likely to have a reportable infection documented. However, the NTDB is a respected and well-regarded database because trained data abstractors are used, and regulatory agencies and insurers use ICD-9. More detailed information, such as Gustilo classifications nor specific surgical treatment rendered such as primary closure, size or extent of soft-tissue injury, or type of flap, are not able to be assessed, so confounders do exist. In addition, diagnostic criteria for each comorbidity and the granular details regarding the severity of the comorbidities, how well the comorbidities are medically controlled, the extent of drug use/smoking are not defined. An issue with the database is that the use of drugs, alcohol, tobacco, etc. are binary responses of yes/no without additional details regarding the levels of use (for example, answering yes to alcohol does differentiate between a patient who drinks one glass of wine per week versus a patient who drinks a liter of liquor daily). Furthermore, the fracture patterns and injury energy levels are not known because of the general grouping of MOIs. Although unlikely given the probability selection, there may be reporting bias regarding which trauma centers are able to participate. Owing to the low outcome results, we could not use logistic regression to determine the independence of predictive variables. Nonetheless, our study used these data consisting of open tibial shaft fracture patients to account for acute inpatient infection related to patient comorbidities from trauma centers across the United States and reached the end points of our study.

The number of patients, over 140,000 open tibial fractures from multiple trauma centers, is a strength of this study. In the field of trauma, population data, such as the NTDB, facilitate evaluation of traumatic injuries beyond case reports, increasing generalizability to improve our understanding of pathology and affect care. In this study, we found an association of patient comorbidities with the development of acute inpatient infection during initial hospitalization for the treatment of open tibial shaft fracture. We found that nonmodifiable patient medical conditions increase the risk of infection in patients with this injury. Treating surgeons can use this to counsel patients and their family regarding inpatient expectations and outcomes. The aggregated national data help eliminate the variability and bias that may be present in studies based on the experience of a single institution and can be used for large-scale modeling to better reflect the patient case-mix index and expected complications.

In conclusion, patient congestive heart failure, bleeding disorders, or obesity are three to 4.5 times more likely to develop an inpatient infection of their open tibial shaft fracture during acute hospitalization than patients without those comorbidities. Patients with diabetes, psychiatric illness, hypertension, or COPD are 1.5 to 2 times more likely to develop subsequent infection compared with patients without those comorbidities. Counseling of patients with these injuries and comorbidities should include discussion of the increased risks. System risk models for the infectious complications after open tibial shaft fractures can be developed to account for these comorbidities. As such, modifiers for these more at-risk patients based on comorbidities should be considered when evaluating surgeon/hospital performance.
